# Supplementary material for: Cortex-wide response mode of VIP-expressing inhibitory neurons by reward and punishment
Source: eLife. 2022 Nov 23;11:e78815. doi: 10.7554/eLife.78815 (PMC9683790; doi:10.7554/eLife.78815)
Supplement: Supplementary file 1. — Scanning speed was calculated according to the equations in the column ‘calculation of scanning speed’. Ratio of collected photons was calculated from relative pixel dwell times. All parameters used for calculations are listed in the bottom field. Note, that chessboard scanning provides 170-fold faster measurement speed and 244-fold higher photon collection compared to volume scanning with resonant mirrors. [file elife-78815-supp1.docx]

|  | schematic of the method | name of the method | calculation of scanning speed | | *T_measurement_* (*v_measurement_* ) | *V_measurement_* compared to chessboard scanning | ratio of collected photons compared to chessboard scanning (*SNR^2^ )* | *SNR^2 ×^ v_gain_* compared to chessboard scanning |
| --- | --- | --- | --- | --- | --- | --- | --- | --- |
| AO SCANNING TECHNIQUES | 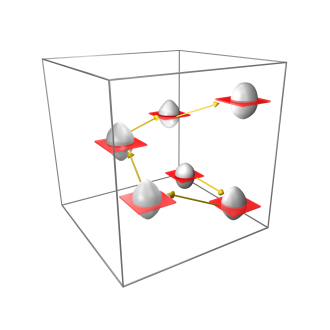 | 3D AO chessboard scanning | ***N_cell_*** *×* ***N_line_*** *×* ***T_pixel_*** | | 0.036 s (27.7 Hz) | 1 | 1 | 1 |
|  | 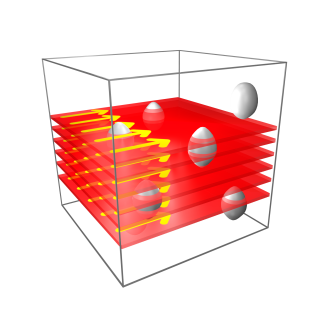 | AO point by point scanning of the entire volume | ***x*** *×* ***y*** *×* ***z*** *×* ***T_pixel_*** | | 1611.5 s (0.0006 Hz) | 1/44762 | 1 | 1/44762 |
|  | 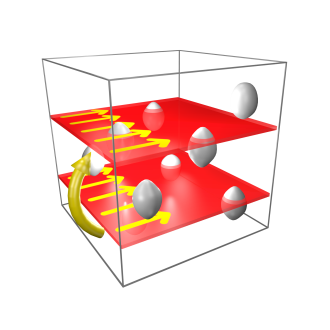 | AO point by point scanning in layers containing cell somatas (19 layers) | ***x*** *×* ***y*** *×* ***N_z_*** *×* ***T_pixel_*** | | 158.4 s (0.00631 Hz) | 1/4399 | 1 | 1/4399 |
| RESONANT SCANNING TECHNIQUES | 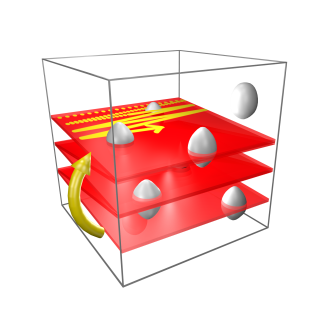 | Volume  scanning  with resonant mirror | ***x*** *×* ***y*** *×* ***z*** *×* ***T'_pixel_*** | | 6.1 s (0.16 Hz) | 1/170 | 1/244 | 1/41506 |
|  | 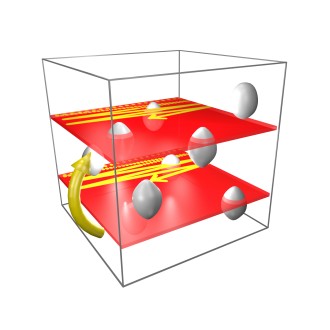 | Multiple-layer scanning with resonant mirror and piezo  (19 layers) | ***x*** *×* ***y*** *×* ***N_z_*** *×* ***T'_pixel_*** | | 0.98 s (1.04 Hz | 1/27 | 1/244 | 1/6654 |
|  | ****Used parameters:*** ***N_cell_*** *= 120 (120 cells)* ***x*** *= 548 pixel,* ***y*** *= 507 pixel,* ***z*** *= 193 pixel (total scanning volume was: x = 689 µm, y = 639 µm, z = 580 µm)*  ***N_z_****=19 (19 z layers were used in volume scanning)* | | | ***T’_pixel_*** *= 0.11 µs, (pixel dwell time of resonant scanning, according to a f=16 kHz frequency and the x=548 pixel line resolution of the resonant scanner)* ***T_pixel_*** *= 30 µs, (AO pixel dwell time)* ***N_line_=*** 10 (number of lines used to form a frame in chessboard scanning) | | | | |
