# Supplementary material for: Cortex-wide response mode of VIP-expressing inhibitory neurons by reward and punishment
Source: eLife. 2022 Nov 23;11:e78815. doi: 10.7554/eLife.78815 (PMC9683790; doi:10.7554/eLife.78815)
Supplement: Supplementary file 2. [file elife-78815-supp2.docx]

| **Animals** | #1 | #2 | | | #3 | | #4 | |
| --- | --- | --- | --- | --- | --- | --- | --- | --- |
| total number of cells recorded | 40 | 40 | | | 39 | | 34 | |
| number of cells with low SNR | 4 | 1 | | | 0 | | 2 | |
| # of cells with low SNR | 1,9,19,20 | 12 | | | 0 | | 9,12 | |
| number of non-responsive cells | 4 | 1 | | | 1 | | 1 | |
| # of cells with no response (#) | 3,10,28,32 | 30 | | | 30 | | 5 | |
| responsive cells | 36 | 39 | | | 39 | | 32 | |
| Ratio of responsive cells (%) | 88.88 | 97.44 | | | 97.44 | | 96.88 | |
| Average ratio (%, mean±SEM) | 95.16±2.09% | |  |  | |  | |  |
